# Limited Chemopreventive Effects of Oral Administration of Polyphenol-60 from Green Tea in the MNU-Induced Rat Mammary Tumor Model

**DOI:** 10.3390/antiox14081009

**Published:** 2025-08-18

**Authors:** Adrian Florin Gal, Dumitrița Rugină, Daria Antonia Dumitraș, Alexandru-Flaviu Tabaran, Maria-Cătălina Matei-Lațiu, Sanda Maria Andrei

**Affiliations:** Faculty of Veterinary Medicine, University of Agricultural Sciences and Veterinary Medicine Cluj-Napoca, 3-5 Mănăștur Street, 400372 Cluj-Napoca, Romania; adrian.gal@usamvcluj.ro (A.F.G.); dumitrita.rugina@usamvcluj.ro (D.R.); antonia.dumitras@usamvcluj.ro (D.A.D.); flaviu.tabaran@usamvcluj.ro (A.-F.T.); sandrei@usamvcluj.ro (S.M.A.)

**Keywords:** chemoprophylaxis, polyphenol-60, green tea, N-methyl-N-nitrosourea, tumorigenesis, mammary cancer, rat

## Abstract

Breast cancer remains one of the most prevalent and lethal malignancies in women and female dogs. Nature offers a plethora of nontoxic medicinal compounds that could be an excellent source of antineoplastic molecules for breast cancer prevention. Due to the closeness of human and rat mammary tumors, one of the best models to study breast cancer is in rats. Accordingly, this study investigated the chemoprophylactic potential of polyphenol-60 (PO-60) from green tea on the mammary tumorigenesis model using female Sprague-Dawley rats. Forty 30-day-old female rats were randomly assigned to four groups (n = 10/group): Group 1 received N-methyl-N-nitrosourea (MNU) intraperitoneally (i.p.), Group 2 received MNU i.p. and 0.5% PO-60 in drinking water, Group 3 received saline i.p. and PO-60, and Group 4 received saline i.p. only. Eventually, rats were subjected to necropsy, histopathology, blood biochemical analysis, and assessment of antioxidative status in liver and mammary tissues. The chronic daily ingestion for 298 days of PO-60 in the MNU-induced mammary tumorigenesis model did not interfere with mammary tumor occurrence and evolution. Still, a decline in GPx and SOD levels in the MNU-inoculated animals (G1/G2 vs. G4) was observed. Catalase activity increased in all groups, except for liver from the individuals inoculated with MNU (G1).

## 1. Introduction

Human breast cancer (HBC) continues to be one of the most common tumors around the globe, with high rates of mortality in women [1]. Similarly, canine mammary cancer (CMC), the most common neoplasia type in female dogs, shares various biological, clinical, pathological, and molecular characteristics with human breast cancer [2,3,4,5]. Although the incidence of and death owing to breast cancer in women have been decreasing since the 1990s, primarily as a result of early identification and treatment protocols in certain nations, the disease’s huge effects have not diminished [6]. Meanwhile, the frequency of mammary cancers in dogs continues to rise, primarily as a result of inadequate early detection measures, most of tumors being diagnosed by accident during a physical exam, and restricted treatment options [5,7]. The primary course of treatment for CMC is surgery, although, up until now, there have been no guidelines for the choice of the kind of surgery for each unique case [8]. Additionally, regarding the use of certain chemotherapeutics, there is a lack of standardization in the selection and use. Establishing these criteria will require more randomized controlled trials and a consensus procedure in addition to developing and assessing novel antineoplastic chemicals that may be less harmful and beneficial to canine mammary cancer patients. The central objective in cancer chemotherapy and/or prevention is the initiation of cell cycle arrest, apoptosis, and suppression of angiogenesis [9,10]. For the time being, to find new complementary or alternative therapies, nature offers a plethora of nontoxic medicinal products that are an excellent source of drugs for cancer prevention and treatment. Accordingly, plant-derived constituents such as polyphenols have proved to have great therapeutic potential by aiming molecular paths of apoptosis [11,12].

Green tea polyphenols have shown notable chemopreventive potential in numerous cancer types [13,14], including melanoma [15,16,17], prostate cancer [18,19,20], lung cancer [21,22], colon cancer [23], skin cancer [24], and triggered apoptosis in human bladder cancer cells [25]. Concerning breast cancer, oral GSP (i.e., Greenselect Phytosome, a lecithin formulation of a caffeine-free green tea catechin extract) increases the bioavailability of EGCG (epigallocatechin-3-*O*-gallate), which is detectable in breast tumor tissue and is correlated with antiproliferative effects on breast cancer tissue [26]. Still, the role of green tea in breast cancer is unclear. Many in vitro and in vivo studies have shown the correlation between green tea intake and the diminished risk of breast cancer. More than ten catechin constituents have been detected in green tea, among which EGCG (epigallocatechin gallate) is the most dominant and has shown the most suppressing effects on breast cancer [27]. Catechins, a group of natural antioxidants, reduce carcinogen-induced ROS and DNA injuries by boosting antioxidant enzymes, supporting the repair of injured DNA, and ROS scavenging. Moreover, catechins (e.g., EGCG) adjust cell signaling pathways involved in breast carcinogenesis, i.e., PI3k/Akt/mTOR, EGFR, ERK, Wnt/β-catenin, and HGF/Met pathways [28,29,30,31]. Furthermore, EGCG interrelates with target proteins (e.g., ERα, Zap-70, PI3K, G3BP1, IGF-1R, vimentin, Bcl-2, Bcl-xL, GRP78, and Fyn via hydrogen bonding) in breast cancerous cells [27,32]. Additionally, catechins prevent DNA methylation by suppressing DNMT (DNA methyltransferase) and increasing SAH (*S*-adenosyl-*L*-homocysteine) levels [33]. By reducing VEGF (vascular endothelial growth factor) promoter activity, EGCG prevents VEGF protein from being secreted, which ultimately prevents tumor angiogenesis. By inducing cell cycle arrest and Ca^2+^-linked apoptosis, promoting TP53/caspase-mediated apoptosis, downregulating anti-apoptotic proteins, inhibiting FAS, and regulating the NO/NOS system, catechins suppress the growth of breast cancer cells and lead them to undergo apoptosis. Through the inhibition of EMT, downregulation of MT1-MMP transcription, and modification of proteolytic enzymes, tea catechins prevent breast cancer cells from dissemination [24,34].

Some other reports have assessed the inhibitory potential of EGCG against breast cancer. Accordingly, EGCG blocks cell cycle progression at the G2/M phase in MCF 7 breast cancer cells and triggers apoptosis by increasing PARP and procaspase expression and impeding miR 25 expression [35]. Unfortunately, there is inconsistency between in vitro and in vivo results obtained from trials, which may be due to the limited oral bioavailability and the unknown behavior of catechins in vivo. To increase oral bioavailability and the anti-tumor effects of tea catechins, new research and techniques for stabilizing catechins in the digestive system would be essential [27].

One of the best models to study breast cancer is on rats due to the closeness of human and rat mammary tumors [12,36]. The elected carcinogenic agent used for mammary tumor induction in rats is *N*-methyl-*N*-nitrosourea (MNU) [12,37,38,39]. Overall, there is a scarcity of data available about the chemopreventive and/or therapeutic potential of molecules found in green tea on in vivo tumorigenesis models. Given this, the current study surveyed the chemoprophylactic effects of the chronic daily intake of polyphenol-60 on MNU-induced mammary carcinogenesis in female Sprague-Dawley rats.

## 2. Materials and Methods

### 2.1. Animals

This research involved 30-day-old female Sprague-Dawley rats (n = 40; 10 rats per group) that were purchased from the Cantacuzino Institute in Romania. Prior to the trial, the rats were housed in conventional laboratory settings, with a temperature of 22–23 °C, a 12 h light–dark cycle, and 60% humidity. With approval from the Animal Ethics Committee of the University of Agricultural Sciences and Veterinary Medicine of Cluj-Napoca (UASVMCN), Romania (UASVM Bio-Ethics Committee No. 9753/22.06.2016), all experiments were carried out in compliance with the European Union’s regulation on animal testing and in accordance with Romanian Animal Research Committee procedures.

### 2.2. Polyphenon-60 (PO-60)

PO-60 (Sigma-Aldrich, Code P1204-25G, Product Number: P1204, CAS Number: 138988-88-2; 3050 Spruce Street, Saint Louis, MO 63103, USA) was diluted in drinking tap water at a dosage of 0.5% and was later administered to the rats. The prepared water with PO-60 was administered ad libitum for 298 days and was replaced every second day. The dose establishment mode was based on other reports suggesting that chronic administration (during 6 months) of green tea polyphenols in drinking water did not induce adverse effects at a dilution of up to 1.5% [40,41].

### 2.3. Experimental Design

Mammary tumors were induced by using MNU as a carcinogenic agent (Sigma-Aldrich Chemical Co., St. Louis, MO, USA). An MNU solution was obtained by dissolving the above-mentioned carcinogen in a standard saline solution [42]. The acquired solution was used immediately after preparation.

The female rats were randomly separated into four groups, and the following treatments were performed:

(a) Rats from Group 1 (G1; n = 10) were intraperitoneally (i.p.) inoculated with a unique dose of 55 mg MNU/kg body weight (BW) and received regular food for rats (supplied by the Cantacuzino Institute, Bucharest, Romania) and tap water.
(b) Rats from Group 2 (G2; n = 10) were inoculated i.p. with a unique dose of 55 mg MNU/kg BW and received regular food for rats and tap water supplemented with 0.5% PO-60, ad libitum.
(c) Group 3 (G3; n = 10) was inoculated i.p. with a unique dose of saline and received regular food for rats and tap water supplemented with 0.5% PO-60, ad libitum.
(d) Group 4 (G4; n = 10) was inoculated with a single dose of saline i.p. and received regular food for rats and tap water.

For stimulating the intracellular antioxidant defense system and ensuring an appropriate antioxidative status of the rat individuals, PO-60 was administrated in tap water starting at the age of 29 days (i.e., 8 days before the administration of the cancer-causing agent). The experiment, including the daily intake of PO-60 (for G2 and G3), continued for 290 days (roughly 9.53 months). Throughout the experiment, a complete survey was performed weekly for each rat, which included, among others, the general body condition, body weight, and analysis of the mammary parenchyma. On welfare grounds, more specifically due to severe threatening body conditions caused by the tumoral progression in some rat individuals, the humane endpoint of the study was established at 290 days (~9.53 months) from MNU administration (or after 298 days of daily ingestion of PO-60).

Eventually, rats from all groups underwent deep narcosis with halothane and humanly exsanguinated later. Several details were considered, i.e., the number of tumoral lesions, incidence, multiplicity, size, and weight of the tumoral lesion. The size of each tumor was assessed using a micrometer caliper.

### 2.4. Necropsy and Histopathology

A comprehensive necropsy examination was realized on the rats used in this study. The gross examination of tumoral lesions detected throughout the body included the following details: tumor location, size, weight, and consistency, along with some details regarding regional lymph nodes (e.g., mobility, hypertrophy). In addition, for each female rat bearing mammary tumors from G1 and G2, the following parameters were calculated:

(a) The percentage of the mammary tumor mass (MTM) compared with the final body weight (FBW) of the rat, using the formula MTM (g) × 100/FBW (g) = MTM (%);
(b) The volume of tumoral mass (MTV) calculated using the formula recommended by Woditschka et al. (2008): 0.5 × tumor width × tumor length × tumor height [43];
(c) Mammary tumor multiplicity (MTMp) for G1 and G2 (i.e., the average number of mammary tumors/rat).

In addition, the necropsy survey included a macroscopic and microscopic examination of all internal organs. For further histological analysis, a variety of tissue samples were collected from several organs, including the lung, stomach, intestine, liver, pancreas, kidneys, spleen, lymph nodes, and central nervous system. After undergoing fixation in 10% buffered formalin, the collected samples were embedded in paraffin. The tissue sections achieved (using a Leica rotary microtome, model RM2125, Nussloch, Germany) were stained using the hematoxylin and eosin (HE) technique. The microphotographs were acquired using an Olympus SC180 Microscope Digital Camera (Tokyo, Japan), along with Olympus cellSens Entry 3.1 (Tokyo, Japan). The mammary tumors were classified as benign or malignant [44]. In the case of the latter, a grading system was utilized [45].

The semi-quantitative tumor score was realized by adopting previously published protocols related to semi-quantitative scores [46,47,48,49]. The tumor-inducing/protection was scored with a semi-quantitative method, assessing the type of neoplasia and the intratumoral inflammation as follows: type of neoplasia: no tumor = 0, non-mammary tumor = 1, benign mammary tumor = 4, malign mammary tumor = 7; intratumoral inflammation: absent = 0, scattered inflammatory cells = 1, diffuse inflammatory reaction = 2. The obtained values for each determination were added up, giving the final score, ranging from 0 to 9.

The biochemical analysis was performed on tissue samples (i.e., liver and mammary tissue) previously collected from the female rats during necropsy.

### 2.5. Assays of Antioxidant Profile in Liver and Mammary Gland

The enzymatic activities of glutathione peroxidase (GPx), superoxide dismutase (SOD), and catalase (CAT), along with the levels of the lipid peroxidation marker malondialdehyde (MDA), were analyzed in liver and mammary tissues of rats. Tissues were carefully sliced and rinsed with ice-cold phosphate-buffered saline (0.9%, pH 7.2). Homogenization was performed using a glass homogenizer in cold PBS, followed by centrifugation at 20,000× *g* for 10 min. The resulting supernatant was collected for enzymatic and oxidative stress assays.

CAT activity was determined using the Catalase Activity Assay Kit (Cayman Chemical, Ann Arbor, MI, USA). The assay relies on the colorimetric detection of formaldehyde generated by catalase, with Purpald (4-amino-3-hydrazino-5-mercapto-1,2,4-triazole) serving as the chromogenic reagent. A standard curve was prepared using bovine liver catalase, and one unit of activity was defined as the amount of enzyme that produces 1 nmol of formaldehyde per minute at 25 °C. Absorbance was measured at 540 nm, and results are expressed as μmol/min/g of tissue.

GPx activity was evaluated using the Ransel kit (Randox Laboratories Ltd., London, UK). The assay is based on the oxidation of NADPH to NADP+ as glutathione disulfide (GSSG) is reduced to glutathione (GSH) by glutathione reductase, following the reaction of GSH with cumene hydroperoxide. The decrease in absorbance at 340 nm was directly proportional to GPx activity.

SOD activity was evaluated using a colorimetric assay (Cayman Chemical, MI, USA). The method is based on the reduction of tetrazolium salt to formazan by superoxide radicals generated in a xanthine/xanthine oxidase system. One unit of SOD is defined as the amount of enzyme needed to produce 50% dismutation of the superoxide radical. Absorbance was measured at 450 nm.

Lipid peroxidation levels were assessed by quantifying malondialdehyde (MDA) levels using the TBARS Assay Kit (Cayman Chemical). MDA reacts with thiobarbituric acid (TBA) under acidic conditions and high temperatures (90–100 °C) to form a colored MDA–TBA adduct, which was detected spectrophotometrically at 540 nm.

All absorbance readings were performed using an HT BioTek Synergy microplate reader (BioTek Instruments, Winooski, VT, USA).

### 2.6. Assays of Blood Biochemical Profile

The animals were weighed before they were put to sleep, and blood samples were collected for further analyses, from the orbital sinus using 10% EDTA (Ethylenediamine Tetra-Acetic Acid) as an anticoagulant. Whole blood was subjected to biochemical analysis using an automated Element RC clinical biochemistry analyzer (Scil Animal Care Company, Altorf, France) to determine the following parameters: total protein, albumin, globulin, alkaline phosphatase (ALP), alanine aminotransferase (ALT), amylase (AMY), calcium (Ca), phosphorus (P), sodium (Na), potassium (K), blood urea nitrogen (BUN), total bilirubin (TBIL), creatinine (CRE), and glucose (GLU).

### 2.7. Statistical Analysis

Statistical calculations were performed using the GraphPad Prism 8 statistics program (San Diego, CA, USA). For pathological and histopathological findings, an unpaired *t*-test was used to assess the differences between the MNU-inoculated groups (in view of MTMp, average MTM relative to FBW (%), MTV, and TNMT-G), with the significance threshold being set at *p* ≤ 0.05. The tumor score results are expressed as median ± IQR (inter-quartile range). Differences between the two MNU-inoculated groups were assessed by the Mann–Whitney U-test, and *p*-values ≤ 0.05 were considered statistically significant.

For biochemical data, one-way and two-way ANOVA tests were performed, and Tukey’s multiple comparisons post hoc test was used, when suitable, to ascertain statistical significance. At a significance threshold of *p* < 0.05, differences between the experimental groups and the control were assessed. The findings are presented as arithmetical mean values ± standard deviations, and each determination was conducted in triplicate.

## 3. Results

### 3.1. Chemopreventive Effects of PO-60 on Mammary Tumor Development

**Figure 1:**
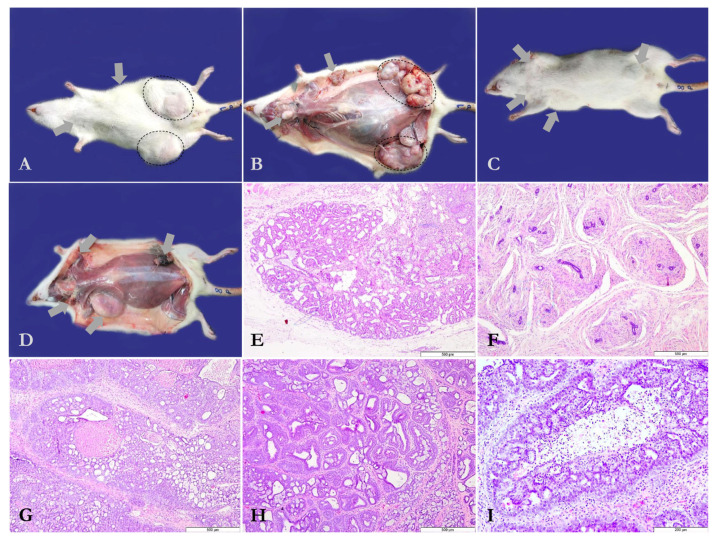
(**A**) Mammary tumors induced by MNU (*N*-Methyl-*N*-Nitrosourea) as subcutaneous nodular structures of different sizes (selected areas and arrows); Rat no. 1, Group 1. (**B**) Macroscopic features of the MNU (*N*-Methyl-*N*-Nitrosourea)-induced mammary tumors following skinning (selected areas and arrows); Rat no. 1, Group 1. (**C**,**D**) MNU (*N*-Methyl-*N*-Nitrosourea)-induced mammary tumors (arrows) in rat individuals from Group 2 (Rat no. 8), including gross features of the tumoral subcutaneous masses. (**E**) In situ cribriform ductal carcinoma, grade 2, represented by numerous luminal cavities with or without secretory product; hematoxylin and eosin stain (Group 1, Rat no. 2, 2nd left mammary gland). (**F**) Fibroadenoma consisting of scattered tubular structures with abundant intertubular fibrous proliferation; hematoxylin and eosin stain (Group 1, Rat no. 3, 2nd left mammary gland). (**G**) Invasive cribriform carcinoma, grade 3, which includes a necrotized area surrounded by numerous tubular structures with a cribriform appearance; hematoxylin and eosin stain (Group 2, Rat no. 1, 5th right mammary gland). (**H**) Invasive tubule-papillary carcinoma, grade 1, composed of large and uneven tubular structures with intraluminal papillary protrusions; hematoxylin and eosin stain (Group 2, Rat no. 1, 5th left mammary gland). (**I**) In situ ductal comedocarcinoma represented by a central necrotized area bordered by tumoral proliferation with secondary lumina; hematoxylin and eosin stain (Group 2, Rat no. 4, 3rd right mammary gland).

Mammary tumors developed only in rat individuals from G1 and G2, i.e., MNU-inoculated rats. Out of 10 rats, 9 (90%) developed mammary tumors in G1 (Figure 1A,B), as compared with G2, in which 7 rats out of 10 (70%) presented mammary tumors (Figure 1C,D). The mammary tumor number per rat ranged from 0 to 9 in G1, whereas a variation of 0 to 7 mammary tumors was observed in rats from G2 (Supplementary Files: Tables S1 and S2). The number of mammary tumors recorded in G1 was 37, while a total of 31 mammary tumors were identified in G2 (Table 1). However, the mammary tumor multiplicity (MTMp) was 3.7 ± 2.7 mammary tumors/rat in G1 and 3.1 ± 2.8 mammary tumors/rat in G2 (*p* > 0.05; Table 1).

Following histological assessment, the mammary tumors detected were categorized as benign (Figure 1F) or malignant lesions (Figure 1E,G,H,I) according to Russo and Russo’s (2000) histologic classification of tumors of the rat mammary gland [44]. As regards the malignancy of the identified mammary tumors in the two MNU-exposed groups, the following differences were observed: 66.6% vs. 76.9% grade I mammary carcinomas (Figure 1H) were noticed in G1 as compared with G2, 28.5% vs. 15.3% grade II mammary carcinomas (Figure 1E) were observed in G1 paralleled to G2, and 4.7% vs. 7.6% grade III mammary carcinomas (Figure 1G) were detected in G1 paralleled to G2. A total of 16% benign mammary lesions were identified in G1 vs. 27.8% in G2 (Figure 1F).

**Figure 2:**
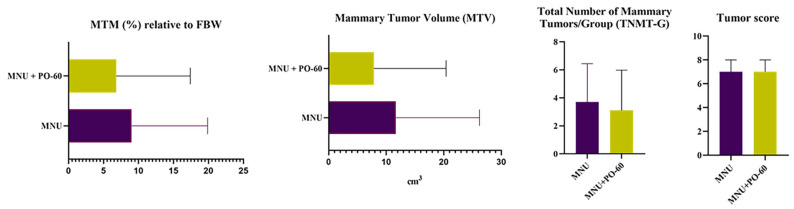
Graphical representation of comparative data between MNU (*N*-Methyl-*N*-Nitrosourea)-exposed groups [Group 1—MNU (*N*-Methyl-*N*-Nitrosourea); Group 2—MNU + PO-60 (*N*-Methyl-*N*-Nitrosourea + Polyphenol-60)]. Values are expressed as mean ± standard deviation (SD) for MTM relative to FBW (%), MTV and TNMT-G (with an irrelevant difference between G1 vs. G2 according to unpaired *t*-test) and as median IQR for tumor score (with an insignificant statistical difference between G1 vs. G2, according to the Mann–Whitney U test).

Microscopically, the identified mammary tumors did not show significant structural variations in the rats from G1 vs. G2. Accordingly, the scoring revealed an insignificant statistical difference between the MNU-inoculated groups, according to the Mann–Whitney U test (Table 1, Figure 2).

However, a limited number of non-mammary tumor types were diagnosed in both MNU-exposed groups (e.g., interstitial renal tumor, leiomyoma, ovarian fibrosarcoma, liposarcoma; Supplementary Files: Tables S1 and S2).

### 3.2. Blood Biochemical Profile in All Experimental Groups

#### 3.2.1. Blood Proteins

**Figure 3:**
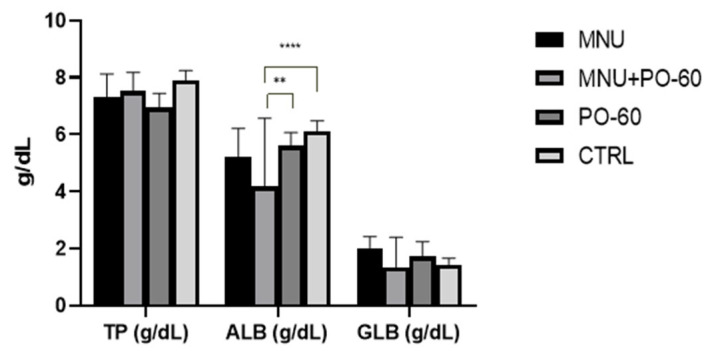
Blood protein profile. Group 1—MNU (*N*-Methyl-*N*-Nitrosourea), Group 2—MNU + PO-60 (*N*-Methyl-*N*-Nitrosourea+Polyphenol-60), Group 3—PO-60 (Polyphenol-60), and Group 4—CTRL (Control). Values are expressed as mean ± SD from three independent experiments. Statistical significance thresholds: ** *p* < 0.01, and **** *p* < 0.0001.

The values of each group were compared to evaluate the parameters. Figure 3 illustrates that there was no significant difference in the concentration of globulins and total protein between the groups. Nonetheless, we observed a statistically significant difference (*p* < 0.01, *p* < 0.0001) in the albumin concentrations between the MNU + PO-60 group (G2) and the MNU-free groups (G2, G3).

#### 3.2.2. The Determination of Antioxidant Enzyme Activity

**Figure 4:**
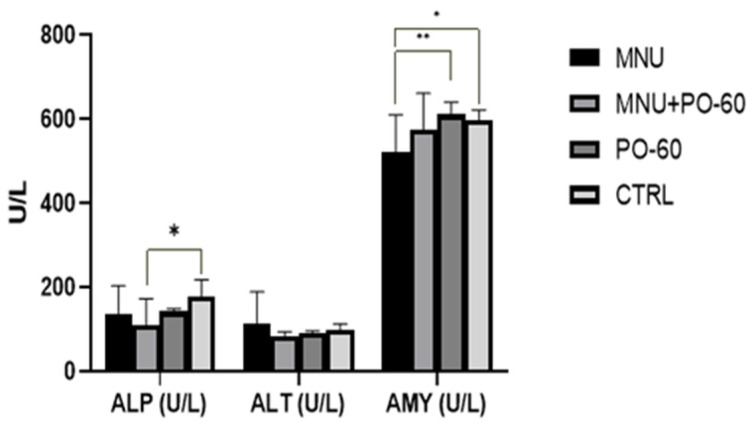
The activity of blood enzymes. Group 1—MNU (*N*-Methyl-*N*-Nitrosourea), Group 2—MNU + PO-60 (*N*-Methyl-*N*-Nitrosourea+Polyphenol-60), Group 3—PO-60 (Polyphenol-60), and Group 4—CTRL (Control). ALP—alkaline phosphatase, ALT—alanine aminotransferase, and AMY—amylase. Values are expressed as mean ± SD from three independent experiments. Statistical significance levels: * *p* < 0.05 and ** *p* < 0.01.

In regard to sanguine enzyme activity, we noticed an increase in the activity of ALT in the MNU-inoculated group as compared with all the other groups, but without statistical significance (G1 vs. G2/G3/G4). ALP activity decreased in the MNU + PO-60-treated group (G2) relative to the CTRL group (G4), exhibiting a statistically significant difference (*p* < 0.05). When compared with the other groups (G3 and G4), the MNU-inoculated group’s (G1, G2) amylase (AMY) activity was lower; however, this difference was only statistically significant when compared with the groups that received PO-60 treatment (*p* < 0.01) and the CTRL group (*p* < 0.05) (Figure 4).

#### 3.2.3. Blood Biochemical Parameters

**Figure 5:**
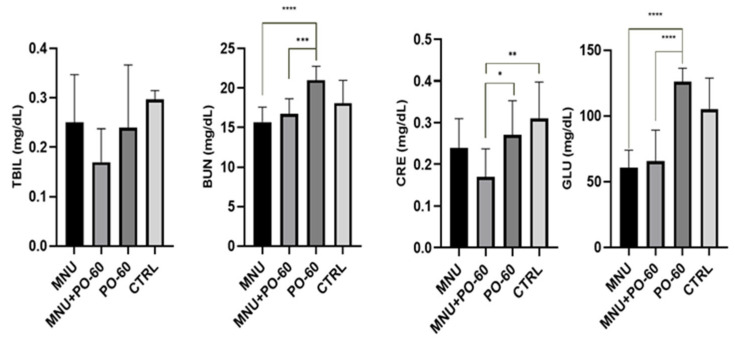
Modulatory influences of MNU and PO-60 on blood biochemical parameters. Group 1—MNU (*N*-Methyl-*N*-Nitrosourea), Group 2—MNU + PO-60 (*N*-Methyl-*N*-Nitrosourea + Polyphenol-60), Group 3—PO-60 (Polyphenol-60), and Group 4—CTRL (Control). TBIL—total bilirubin, BUN—blood urea nitrogen, CRE—creatinine, and GLU—glucose. Values are expressed as mean ± SD from three independent experiments. Statistical significance levels: * *p* < 0.05, ** *p* < 0.01, *** *p* < 0.001, and **** *p* < 0.0001.

The administration of MNU and PO-60 did not show any statistical change in the TBIL concentration. However, we observed a slight decrease in TBIL concentration following the MNU + PO-60 group (G2), compared with the non-treated individuals (*p* > 0.05). BUN concentration was statistically higher in the case of individuals treated with PO-60 (G3) compared with the case of those belonging to G1 (*p* < 0.0001) and G2 (*p* < 0.001). A statistically significant decreased level of CRE was noticed following MNU and PO-60 treatment (G2) as compared with MNU-free groups G3 (*p* < 0.05) and G4 (*p* < 0.01), whereas MNU-treated groups (G1, G2) displayed lower levels of GLU compared with the individuals treated with PO-60 (G3; *p* < 0.0001; Figure 5).

#### 3.2.4. Blood Microminerals

**Figure 6:**
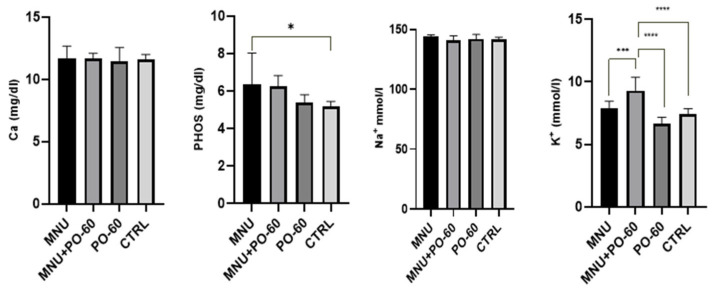
Modulatory influences of MNU (*N*-Methyl-*N*-Nitrosourea) and PO-60 (Polyphenol-60) on blood microminerals. Group 1—MNU (*N*-Methyl-*N*-Nitrosourea), Group 2—MNU + PO-60 (*N*-Methyl-*N*-Nitrosourea + Polyphenol-60), Group 3—PO-60 (Polyphenol-60), and Group 4—CTRL (Control). Ca—calcium, PHOS—phosphorus, Na^+^—sodium, and K^+^—potassium. Values are expressed as mean ± SD from three independent experiments. Statistical significance levels: * *p* < 0.05, *** *p* < 0.001, and **** *p* < 0.0001.

The concentration of the tested microminerals was influenced by the MNU inoculation and the PO-60 treatment, as can be seen in Figure 6. As a result, calcium and sodium concentrations were not affected, while phosphorus concentrations were statistically higher (*p* < 0.05) in G1 vs. G4. Potassium levels were the ones most affected as follows: treatment with PO-60 of the MNU-inoculated individuals (G2) led to an increased concentration as compared with all the other groups (*p* < 0.001). However, the PO-60-treated individuals (G3) showed the lowest level of potassium compared with both MNU-inoculated (G1; *p* > 0.05) and MNU+PO-60 individuals (G2; *p* < 0.0001).

### 3.3. The Effects of MNU and PO-60 on the Antioxidative Status in Tissues

#### 3.3.1. Antioxidant Defense System in Rat Liver

**Figure 7:**
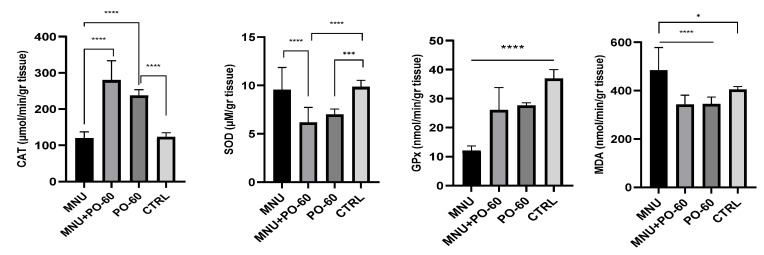
Effect of MNU (*N*-Methyl-*N*-Nitrosourea) inoculation and PO-60 (Polyphenol-60) administration on hepatic antioxidative enzymatic defense system in rats. Group 1—MNU (*N*-Methyl-*N*-Nitrosourea), Group 2—MNU + PO-60 (*N*-Methyl-*N*-Nitrosourea + Polyphenol-60), Group 3—PO-60 (Polyphenol-60), and Group 4—CTRL (Control). CAT—catalase, SOD—superoxide dismutase, GPx—glutathione peroxidase, and MDA—malondialdehyde. Values are expressed as mean ± SD from three independent experiments. Statistical significance levels: * *p* < 0.05, *** *p* < 0.001, and **** *p* < 0.0001. Comparisons for the antioxidant profile were made based on the one-way ANOVA, followed by Tukey’s test (GraphPad Prism version 8).

Concurrently, superoxide dismutase (SOD), catalase (CAT), glutathione peroxidase (GPx), and malondialdehyde (MDA) measurements in the liver were recorded. The MNU inoculation led to a slight decrease in catalase and superoxide dismutase as compared with the CTRL group (G1 vs. G4), whereas GPx activity dropped significantly (*p* < 0.0001). Regarding MDA, we noticed a significant increase following the administration of MNU compared with the CTRL group (G1 vs. G4, *p* < 0.0001). In relation to the CTRL group (G4), in most of the other groups, the administration of PO-60 led to a decrease in antioxidant enzymes (G4 vs. G2 and G3), except for CAT. This is the case of SOD (*p* < 0.001), GPx (*p* > 0.05), and MDA (*p* > 0.05). Following PO-60 treatment (in G2 and G3), CAT was the only antioxidant enzyme to exhibit enhanced activity (G2 vs. G4, *p* > 0.05). No discernible changes in antioxidant activity were noticed between the MNU-inoculated group (G1) and the MNU + PO-60-treated group (G2), except for CAT (which expressed lower levels in G1 vs. G2; *p* < 0.0001) and SOD, which showed increased levels in G1 vs. G2 (*p* < 0.0001; Figure 7).

#### 3.3.2. Antioxidant Defense System in Rat Mammary Gland

**Figure 8:**
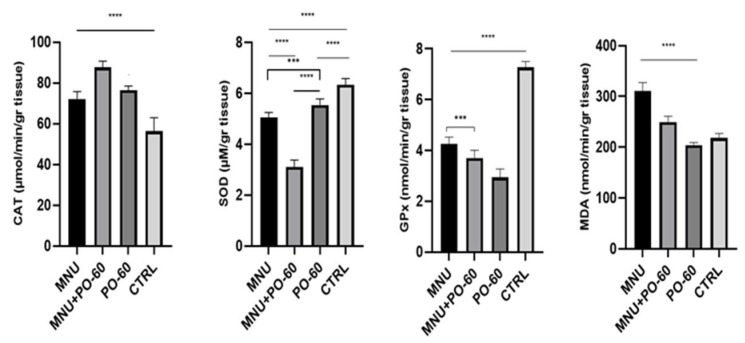
Effect of MNU (*N*-Methyl-*N*-Nitrosourea) inoculation and PO-60 (Polyphenol-60) administration on antioxidative markers in the mammary gland. Group 1—MNU (*N*-Methyl-*N*-Nitrosourea), Group 2—MNU + PO-60 (*N*-Methyl-*N*-Nitrosourea + Polyphenol-60), Group 3—PO-60 (Polyphenol-60), and Group 4—CTRL (Control). CAT—catalase, SOD—superoxide dismutase, GPx—glutathione peroxidase, and MDA—malondialdehyde. Values are expressed as mean ± SD from three independent experiments. Statistical significance levels: *** *p* < 0.001, and **** *p* < 0.0001. Comparisons for the antioxidant profile were made based on the one-way ANOVA, followed by Tukey’s test (GraphPad Prism version 8).

When compared with the CTRL group, it was noticed that the MNU administration increased the activities of CAT (G1 vs. G4, *p* < 0.0001; G2 vs. G4, *p* > 0.05) and MDA (G1 vs. G4, *p* > 0.05). On the other hand, following MNU inoculation (G1), SOD and GPx decreased their activities significantly as compared with the CTRL group (G1 vs. G4, *p* < 0.0001). The PO-60 administration in the diet of rats from G2 determined a substantial drop in the activity of SOD, with statistical significance recorded in G2 vs. G3 (*p* < 0.0001), whereas GPx recorded a drop in G2 vs. G4 (*p* > 0.05). CAT and MDA increased in G2 vs. G4 (*p* > 0.05; Figure 8).

## 4. Discussion

Green tea is a widely consumed beverage worldwide and contains constituents (e.g., catechins) that are thought to be very effective in the chemoprevention and treatment of a variety of diseases. PO-60 from green tea is an assortment of catechins, including epigallocatechin gallate (EGCG), epicatechin gallate (ECG), epigallocatechin (EGC), and epicatechin (EC). It acts as a strong antioxidant by scavenging free radicals and implicitly oxidative stress reduction. In essence, the action mechanism of PO-60 includes ROS activity inhibition and modulation of cellular signaling pathways associated with oxidative damage [50,51].

Past investigations have confirmed that green tea and its primary constituent, epigallocatechin gallate (EGCG), have a potential role in the management of cancer. However, despite the notable antitumor activities of tea catechins, some studies have proved a limited pharmacological application in clinical settings as a result of their poor in vivo stability, degradation in the gastrointestinal tract, limited bioavailability, and insufficient intestinal absorption [52]. Accordingly, polyphenon-E (PE) raised the death ratio of lung cancer cells by inhibiting Bcl-2-related apoptosis by reducing the membrane potential of mitochondria and boosting PARP cleavage [52,53]. Likewise, PE suppressed the growth of colon cancer cells [51]. In another report, PE triggered severe and persistent stress on the endoplasmic reticulum in prostate cancer cells that induced cell death through a caspase-independent necroptosis [54]. Other studies have shown that tea polyphenols induce apoptosis in mouse skin tumors and human fibrosarcoma HT-1080 cells, through intrinsic cell death pathways [55,56]. Similarly, Epigallocatechin-3-gallate triggers apoptosis in estrogen receptor-negative human breast carcinoma cells through modulation in the protein expression of Bax, p53, and caspase 3 activations [57].

Angiogenesis, an essential process for tumor development and expansion, was reduced by CSNPs-PE in Ehrlich tumors in mice [52]. The anti-angiogenic effect of tea catechins was represented by a decrease in VEGF synthesis, a reduction in VEGF binding to corresponding receptors, and the inhibition of VEGF receptor phosphorylation. Consequently, CSNPs-PE demonstrated the anti-angiogenic effect by inhibiting VEGF and CD31, which prevents the interaction between the tumor and its microenvironment and eventually inhibits tumor growth and expansion [58,59].

Some recent meta-analyses involved seven reports of breast cancer incidence (i.e., two cohort studies and five case-control studies) and two investigations of breast cancer recurrence [60,61,62,63]. Concerning the breast cancer incidence studies, a statistically significant reduction of 19% was observed in women with high green tea consumption. Case–control surveys indicated a similar effect as the overall analysis, i.e., a 19% decrease in risk in green tea drinkers. Nevertheless, in separately analyzed cohort studies, no association between green tea consumption and breast cancer incidence was observed, as demonstrated in the present study. However, the two cohort studies that evaluated the risk of breast cancer recurrence in relation to green tea ingestion showed that high ingestion of green tea may be correlated with a decline in the relative risk in stage I and II breast cancer recurrence [6,64,65,66]. In conclusion, epidemiological surveys regarding the link between green tea and breast cancer continue to be unconvincing [6].

To date, various studies have investigated the therapeutic effects of green tea on breast cancer using rodent models. A variety of green tea products, mixtures, or specific catechins have been utilized [50]. One of the studies performed in female mice demonstrated that treatment with EGCG at a dose of 50 to 100 mg/kg per day in drinking water significantly inhibited the progression of breast cancer. Another report suggested that the influence of EGCG on tumor size was mediated by the inhibition of HIF-1α (hypoxia-inducible factor 1α) and NFκB (nuclear factor κB) activation along with VEGF (vascular endothelial growth factor) expression [6,67,68].

In the present report, despite observing a drop in MTMp value from 3.7 ± 2.7 in G1 to 3.1 ± 2.8 in G2, along with a lower average MTM relative to FBW (9.0 ± 10.8 in G1 vs. 6.8 ± 10.5 in G2), inferior average MTV (16.6 ± 33.3 in G1 vs. 13.8 ± 44.6 in G2), and smaller total number of mammary tumors/group in G2 vs. G1 (i.e., 37 vs. 31), none of the above-mentioned parameters were statistically significant (*p* > 0.05).

In terms of the blood’s biochemical profile, the administration of MNU resulted in a modest decrease in globulins (G2 vs. G4) and total blood proteins (G1 and G2 vs. G4). Albumins decreased slightly following MNU inoculation (G1 vs. G4) and also following MNU inoculation associated with PO-60 treatment (*p* < 0.0001), in comparison with the control group (G2 vs. G4). Additionally, a statistically significant difference (*p* < 0.01) in albumin concentration was observed between the MNU-PO-60 group (G2) and the PO-60 group (G3). The slight reduction in total blood proteins can be a consequence of proteolysis. Proteins can directly undergo hydrolytic deterioration by protein oxidation, or indirectly through increased vulnerability to proteolytic enzymes. It was demonstrated that malignant development is associated with both pericellular and intracellular digestion of proteins by human breast cancer cells [69].

Rats exposed to MNU may have developed hypoalbuminemia as a result of the substance’s capacity to harm liver polyribosomes and components of the cell sap. Alkylation and carboxylation of proteins and RNA may be the source of the damage, although RNA has the highest amount of methyl groups (70–80%) that are transported to polyribosomes [70].

The blood enzymes’ activity was slightly decreased by the MNU inoculation, with amylase showing the most significant drop (G1 vs. G3/G4; *p* < 0.05). A significant drop in ALP activity (*p* < 0.05) was observed in the MNU-PO-60 group (G2) as compared with the CTRL group (G4). Furthermore, there were no significant alterations in the enzymatic profile following the PO-60 administration. Interestingly, concerning the alkaline phosphatase (ALP) activity in patients with cancer, Shreya et al. (2023) [71] observed that 41% of the patients with early-stage breast cancer had elevated ALP levels, as well as 59% of patients with advanced-stage breast cancer. It seems that progressive increase in ALP level along with cancer stage development is an indicator of metastasis, whereas high ALP predicts metastasis in the bone and liver [71].

Overall, the blood biochemical parameters (TBIL, BUN, CRE, and GLU) were slightly decreased by the inoculation of MNU, with statistical significance being observed in BUN (G1/G2 vs. G3; *p* < 0.0001 and *p* < 0.01, respectively), CRE (G2 vs. G4), and GLU (G1/G2 vs. G3; *p* < 0.0001). Most of the blood microminerals were not influenced by both MNU inoculation and PO-60 administration, except for potassium, which significantly increased as compared with both the PO-60 and CTRL groups (G2 vs. G2/G3; *p* < 0.0001). The elevated potassium levels conflicted with most of the other polyphenolic compound findings from earlier research, with only one exception [72]. Given that potassium excretion is mostly carried out by the kidneys, the extraordinarily high levels detected in our study raise questions about renal flux efficiency. Initial hyperkalemia is similar to early renal dysfunction in that it can appear even when the levels of sodium, calcium, and chloride are within normal limits. Such changes call for close observation and more studies to determine any possible effects on cardiovascular and renal health. Hyperkalemia can be pervasively observed in primary and/or metastatic diseases that disturb the adrenal axis. It can also develop as a consequence of immunotherapy, which can cause adrenalitis or hypophysitis [73].

The ability of endogenous antioxidant enzymes to function effectively as the first line of defense against reactive oxygen species (ROS) indicates that these enzymes are useful for assessing the potential extent of oxidative damage caused during carcinogenesis. Depending on the concentration, ROS influences cancer progression in apparently differing ways, either initiating and stimulating tumorigenesis and supporting the transformation and proliferation of cancer cells or triggering cell death [73]. According to the present study, both in the liver and mammary gland, SOD concentrations were decreased in all the MNU-exposed individuals compared with the CTRL or PO-60 groups (G4 or G3), especially in the tumor-bearing individuals treated with PO-60 (G2 vs. G4 in the liver, G2 vs. G3 in the mammary gland; *p* < 0.0001), which may be related to the decline in antioxidant status brought on by mammary carcinogenesis. Catalytic antioxidants that scavenge O^2−^ (singlet oxygen) include cytosolic copper/zinc superoxide dismutase (CuZnSOD, or SOD1), mitochondrial manganese MnSOD (SOD2), and extracellular EC-SOD (SOD3), each of which catalyzes the conversion of O^2−^ to H_2_O_2_ and O_2_. SOD1 and SOD2 offer protection against spontaneous tumorigenesis, and they may be upregulated during tumorigenesis [74].

The decline in GPx level mainly in the MNU-inoculated animals (G1, G2) as compared with the CTRL group (G4) could have the same cause, significant drops of GPx levels being detected only in the G1 vs. G4 groups (*p* < 0.0001). Catalase activity was increased in all the study groups, both in the liver and mammary tissue, except for the liver from the individuals inoculated with MNU (G1). A significant increase in CAT levels in the mammary parenchyma was observed in G1 vs. G4 (*p* < 0.0001), along with a significant drop in CAT levels in G1 vs. G3 in the liver (*p* < 0.0001). ROS activity at the cellular level may account for the elevated CAT value in tumoral cells or in the plasma of tumor-bearing patients. Overall, enzymes that scavenge H_2_O_2_ include catalase (CAT), which converts H_2_O_2_ to H_2_O and O_2_, and glutathione peroxidases (GPXs), which reduce H_2_O_2_ to H_2_O [74,75]. Neoplastic cells constantly adjust to their surroundings by rewiring their metabolism to provide the energy needed for both growth and survival. However, an enormous build-up of ROS can cause severe cell damage, which will ultimately lead to cell death, even in neoplastic cells. Tumor cells contain defense mechanisms that allow them to adapt to oxidative stress by increasing their antioxidant response. This keeps the amount of reactive oxygen species (ROS) in the tumor below the critical threshold while still being greater than in normal cells [76].

The MDA levels were increased in the samples collected from the MNU-inoculated rats, both in the liver and breast tissue, a significant increase in MDA levels being noticed in the liver in G1 vs. G3\G4 (*p* < 0.0001 and *p* < 0.05, respectively) and mammary parenchyma in G1 vs. G3 (*p* < 0.0001). As compared with high MDA hepatic levels in G1, nonsignificant (*p* > 0.05) lower levels of MDA were observed following the MNU-PO-60 administration in G2 individuals as compared with the CTRL group (G4). Malondialdehyde (MDA) is a highly reactive byproduct of lipid peroxidation and a marker of oxidative stress, which may alter the structure of the cellular plasma membrane and may later cause DNA alteration in affected cells. Similar to our findings, in some other reports, the levels of MDA were significantly higher in patients with breast and lung cancer, meningiomas, and gliomas compared with healthy controls [77,78].

## 5. Conclusions

Recently, the body of literature has been focusing on the use of natural products for cancer prevention, but few trials have validated undeniable benefits. Despite promising results shown by some natural compounds, no natural agent has been confirmed to be efficient against all cancer types. Concerning mammary cancer, the ingestion of some dietary natural products is regarded to be inversely associated with their occurrence by epidemiological and experimental reports. The best approach for breast cancer research is employing experimental animals as they are more reliable than in vitro tests by avoiding the absence of biokinetics and data misinterpretation. Still, the appraisal of long-lasting, day-to-day administration of natural products with presumed antitumor effects on animal models is scarce. Considering this, in our survey, the chronic daily ingestion for 298 days of PO-60 from green tea in MNU-induced mammary carcinogenesis in female Sprague-Dawley rats provided perplexing pathological and biochemical results. As in the case of other natural products, the findings could be attributable to the low in vivo bioavailability and stability, degradation in the gastrointestinal tract, and/or insufficient intestinal absorption. Finally, to overcome these limitations, a sustained future research endeavor is required equally to increase the bioavailability and analyze the intricate action mechanisms of polyphenon-60 in mammary cancer prevention and treatment.

## Figures and Tables

**Table 1:** Comparative data concerning mammary and non-mammary tumors developed in rats from Groups 1 and 2.

Experimental Group	MTMp ^1^	Average MTM ^2^ Relative to FBW ^3^ (%)	Average MTV ^4^	TNMT-G ^5^	Non-Mammary Tumors/Group	Semi-Quantitative Tumor Scores (Median IQR), Range 0–9
Group 1	3.7 ± 2.7	9.0 ± 10.8	16.6 ± 33.3	37	4	7 (1–9)
Group 2	3.1 ± 2.8 ^ns1^	6.8 ± 10.5 ^ns1^	13.8 ± 44.6 ^ns1^	31 ^ns1^	5	7 (0–9) ^ns2^

^1^ MTMp—mammary tumor multiplicity (i.e., average mammary tumor number/rat); ^2^ MTM—mammary tumor mass; ^3^ FBW—final body weight; ^4^ MTV—mammary tumor volume calculated using the formula suggested by Woditschka et al., 2008 [43]; ^5^ TNMT-G—total number of mammary tumors/group; ^ns1^—*p* > 0.05 for unpaired *t*-test; ^ns2^—*p* = 0.5149 for Mann–Whitney test.

## Data Availability

Data are contained within the article.

## Supplementary Materials

The following supporting information can be downloaded at https://www.mdpi.com/article/10.3390/antiox14081009/s1: Table S1: Mammary tumors detected in Group 1: tumor location, size, histological features, and other non-mammary tumors identified; Table S2: Chemopreventive effects of PO-60 on mammary tumor occurrence in Group 2: tumor location, size, histological features, and other non-mammary tumors identified.
