# Supplementary material for: Limited Chemopreventive Effects of Oral Administration of Polyphenol-60 from Green Tea in the MNU-Induced Rat Mammary Tumor Model
Source: Antioxidants (Basel). 2025 Aug 18;14(8):1009. doi: 10.3390/antiox14081009 (PMC12382878; doi:10.3390/antiox14081009)
Supplement: Supplementary file 1 [file antioxidants-14-01009-s001.zip › antioxidants-3723739-supplementary.pdf]

Supplementary files

Table S1. Mammary tumors detected in Group 1: tumor location, size, histological features, and other non-mammary tumors identified.

Mammary tumor type	Group 1/Rat no.									
	1	2	3	4	5	6	7	8	9	10
Fibroadenoma	-	-	M2L, I- (5/4.5 cm)	-	M4R, I- (1.7/1.4 cm)	-	-	-	-	M1L, I- (1.4/1.2 cm)
Adenoma (tubulary)	-	-	-	-	-	-	M2R, I- (1.1/1 cm) M2L, I+ (1/0.7 cm)	-	-	-
In situ papillary ductal carcinoma	-	-	M3L, G1, I- (6.5/5.1 cm)	M4L, G1, I++ (5.4/3.2 cm)	-	-	-	-	-	-
In situ solid ductal carcinoma	-	-	M4R, G1, I- (1-2.2 cm)	M2R, G3, I+ (0.8/0.5cm)	-	-	-	-	-	-
In situ cribriform ductal carcinoma	-	M2R, G1, I- (2.2/1.3 cm) M2L, G2, I++ (2.4/2.2 cm)	M1R, G1, I- (3/2.5 cm)	M5R, G1, I++ (7.9/6.5 cm)	-	M1R, G2, I++ (0.8/0.6 cm)	-	-	-	M5L, G1, I- (1.2/1 cm)
In situ ductal comedocarcinoma	-	-	-	M2L, G2, I++ (3/2.7 cm)	-	-	-	-	-	-
Invasive tubular carcinoma	-	M3R, G2, I++ (6.0/3.5 cm)	-	-	-	-	M1R, G1, I+ (1.2/0.5 cm)	-	-	-
Invasive tubule-papillary carcinoma	M1R, G1, I- (1.3/1.3 cm) M2L, G1, I- (1.3/2.1 cm)	M5R, G1, I+ (1.7/1.2 cm) M3L, G1, I+ (2.1/2.0 cm)	M3R, G1, I- (3.5/3 cm) M5R, G1, I- (1.3/1.2 cm)	-	-	M3L, G2, I++ (4.2/3.7 cm)	M3L, G1, I+ (0.9/0.9 cm) M4L, G1, I- (0.9/0.6 cm)	M2R, G2, I- (1.3/1.2 cm) M2L, G1, I+ (2.8/2.3 cm)	-	M3R, G2, I+ (1.2/1.2 cm)

	M5L, G1, I- (6.8/3.5 cm)	M5L, G1, I+ (1.3/0.9 cm)	M1L, G1, I+ (1/2 cm)<br>M4L, G1, I- (6.2/5.6 cm)<br>M5L, G1, I+ (4/3.5 cm)							
Invasive cribriform carcinoma	-	M1R, G1, I+ (1/1.4 cm)	-	-	-	-	-	-	-	-
Carcinosarco-ma	M4R, G1, I- (3.5/2.6 cm)	-	-	-	-	-	-	-	-	-
**Average MTM relative to FBW (%)**	17.39	10.78	29.27	23.87	0.25	4.76	0.97	1.82	0	0.95
**Other tumor types**	-	Interstitial renal tumor	-	-	-	-	-	-	Ovarian fibro-sarcoma<br>Lipo-sarcoma	-

MTM – Mammary Tumour Mass; FBW – Final Body Weight; M_{1-5} – mammary gland number, respective its side (i.e., right - R, left -L) and size (cm); G – Histological grade; I – intratumoral inflammation (absent -, scattered inflammatory cells +, and diffuse inflammatory reaction ++)

Table S2. Chemopreventive effects of PO-60 on mammary tumor occurrence in Group 2: tumor location, size, histological features, and other non-mammary tumors identified.

**Mammary tumor type**	**Group 2/Rat no.**									
	**1**	**2**	**3**	**4**	**5**	**6**	**7**	**8**	**9**	**10**
Fibroadenoma	-	-	-	-	-	-	M3L, I- (2.1/1.5 cm)	M3R, I- (4.8/4 cm)	-	-
Adenoma (tubulary)	M2R, I- (0.6/0.5 cm)	-	-	-	-	-	-	-	-	-
Adenoma (lacting)	-	-	-	-	M3L, I- (3.3/3.4 cm)	-	-	-	-	-
Papillary cystadenoma	-	-	-	-	M4R, I- (0.7/0.5 cm)	-	-	-	-	-
In situ ductal comedocarcino-ma	-	-	-	M3R, G1, I++ (3.5/2.7 cm)	-	-	-	-	-	-
Invasive tubular carcinoma	-	M5R, G1, I+ (1.5/1.7cm)	-	M1R, G3, I++ (8/8 cm)	-	-	-	-	-	-
Invasive tubule-papillary carcinoma	M1L, G1, I- (1/0.7 cm) M4L, G2, I- (5.4/2.1 cm) M5L, G1, I- (4/3.5 cm)	-	M3R, G1, I+ (3/2.7 cm) M5R, G1, I+ (2.5/1.5 cm) M4L, G1, I+ (1/1.2 cm)	M2L, G2, I++ (1.2/1cm) M4L, G2, I+ (2/1.3 cm)	M1R, G1, I+ (1.5/1.3 cm) M3R, G1, I+ (2/2.1 cm) M1L, G1, I- (1.5/1 cm)	-	M2L, G1, I+ (1.3/1.2 cm)	M1R, G1, I- (1.5/1.1cm) M1L, G1, I- (0.7/0.6 cm) M4L, G1, I++ (2.5/1.8cm)	-	-
Invasive cribriform carcinoma	M1R, G1, I+ (1/0,6 cm) M5R, G3, I+ (6/4,5 cm)	-	M2R, G1, I- (1,5/1,4 cm)	M4R, G1, I++ (3.1/2 cm) M1L, G1, I++ (4,1/1,3 cm) M3L, G2, I+ (1.4/1cm)	M2L, G2, I+ (1/0.7 cm) M4L, G1, I++ (1.7/1 cm)	-	-	-	-	-
**Average MTM relative to FBW (%)**	15.57	3.28	3.18	33.78	4.71	0	1.14	6.57	0	0

**Other tumor types**	Interstitial renal tumor	Interstitial renal tumor	-	Mammary metastases in the lung	-	-	-	Interstitial renal tumor	Leio-myoma	-

MTM – Mammary Tumour Mass; FBW – Final Body Weight; M_{1-5} – mammary gland number, respective its side (i.e., right - R, left -L) and size (cm); G – Histological grade; I – intratumoral inflammation (absent -, scattered inflammatory cells +, and diffuse inflammatory reaction ++)
